# Dual Use of Cannabis with Tobacco Is Associated with Increased Sugary Food and Drink Intake in Young People

**DOI:** 10.3390/ijerph21081016

**Published:** 2024-08-02

**Authors:** Niamh Malhotra, Nikita Kasaraneni, Zoya Ahadian, Howard Chang, Ira Advani, Jade McDermott, Caitlyn Truong, Samvel Gaboyan, Ankita Mittal, Alexia Perryman, Jorge A. Masso-Silva, Christine M. Steeger, Russell P. Bowler, Peter J. Castaldi, Sunita Sharma, Laura E. Crotty Alexander

**Affiliations:** 1 Division of Pulmonary, Critical Care, Sleep and Physiology, University of California San Diego, San Diego, CA 92093, USAnkasaran@ucsd.edu (N.K.); zoyaahadian@gmail.com (Z.A.); c2truong@ucsd.edu (C.T.); sgaboyan@ucsd.edu (S.G.); anperryman@health.ucsd.edu (A.P.); jmassosilva@health.ucsd.edu (J.A.M.-S.); 2 Pulmonary Critical Care Section, VA San Diego Healthcare System, San Diego, CA 92161, USA; 3 Institute of Behavioral Science, University of Colorado Boulder, Boulder, CO 80309, USA; christine.steeger@colorado.edu; 4 Department of Genomic Sciences and Systems Biology, Lerner Research Institute, Cleveland Clinic, Cleveland, OH 44195, USA; russellbowler@icloud.com; 5 Channing Division of Network Medicine, Brigham and Women’s Hospital, Harvard Medical School, Boston, MA 02115, USA; repjc@channing.harvard.edu; 6 Division of Pulmonary Sciences and Critical Care Medicine, Department of Medicine, University of Colorado Anschutz School of Medicine, Aurora, CO 80045, USA; sunita.sharma@cuanschutz.edu

**Keywords:** marijuana, THC, exercise, diet, tobacco, nicotine, e-cigarettes, teenagers and young adults

## Abstract

Rates of cannabis initiation among teenagers and young adults are increasing. Further, the use of various forms of cannabis (smoked or vaped) with nicotine (dual use) is increasingly common among young people. The health effects of dual use are lesser known, particularly in the context of high-potency cannabis products and across different routes of administration, which is ominous in terms of predicting future health outcomes. There is a long history of cannabis use being associated with decreased activity and increased snacking, both of which could portend an increased risk of metabolic and cardiovascular disease, particularly when these habits begin during formative years. However, modern forms of cannabis may not have these same effects. Here, we assess whether cannabis use alone and dual use of cannabis with nicotine impact dietary and exercise habits in young people. An anonymous, social media-based survey was designed based on the UC San Diego Inhalant Questionnaire and published diet and exercise questionnaires. A total of 457 surveys were completed. Young sole cannabis users represented 29% of responders, 16% were dual users of cannabis and nicotine, and 55% were non-users of either drug. Although the sole use of cannabis was not associated with dietary or activity differences relative to non-users, dual users of cannabis and nicotine reported higher consumption of unhealthy sugars. This novel finding of dual use being associated with increased sugar intake in young people raises concerns for an increased risk of metabolic syndrome and cardiovascular disease in this population.

## 1. Introduction

Given the recent legalization of cannabis in many states, the prevalence of its use has significantly increased among young people, with a staggering 245% increase in reported cases of abuse and misuse from 2000 to 2020 [1]. In particular, vaping tetrahydrocannabinol (THC) is increasing in popularity among young people, whereas other forms of cannabis consumption are declining in this group [2]. Cannabis use, including smoking marijuana, has anecdotally been associated with poor dietary habits, including binge eating, often referred to as “munchies” [3], and decreased physical activity [4]. Cannabis may also be associated with additional poor health practices due to the attitudes and behaviors associated with psychoactive or illicit drug use [5]. However, cannabis products have evolved drastically over the last few decades, such that the active and inactive ingredients across products are highly varied [6,7]. It is possible that smoking cannabis many years ago did cause unhealthy dietary and activity habits, whereas vaping THC, ingesting edibles, or smoking newer strains may not have the same effect [8,9]. Further, current popular strains of cannabis used in combustible products have different levels of active chemicals, such as Δ-8, Δ-9, and Δ-10-tetrahydrocannabinol, relative to historic strains, and vaped THC has different chemical levels relative to smoked forms of cannabis [10,11,12]. Overall, there is high variability in potency across cannabis products, and different products may have differing effects on addiction and mental and physical health outcomes [13]. We hypothesized that vaping THC would not lead to decreased activity but would be associated with unhealthy eating habits.

For many years, the use of combustible tobacco has declined among teenagers and young adults, from rates of 29.1% in 1997 to 5.4% in 2020 [14]. However, the rise of vaping nicotine (a form of tobacco) has blunted the overall decline in tobacco use [14]. Rates have been climbing again among youth, with 12.6% of high schoolers currently using tobacco products [15]. E-cigarettes are the most popular tobacco product, with 10% of high schoolers actively vaping nicotine [15]. We and other groups have found high rates of dual use of nicotine and cannabis products, both in young people and in adults [16,17]. Additionally, dual use is higher in lesbian, gay, bisexual, transgender, queer or questioning, intersex, asexual, and other identities ([18] IA+) and Black, Indigenous, and People of Color (BIPOC) communities [19,20]. In contrast to cannabis, nicotine has been shown to have appetite suppressing effects [21,22]. Because studies have shown that nicotine and cannabis use habits and addiction often develop simultaneously among young people [23], there may be interactions between tobacco and cannabis products and their effects on diet and exercise. Further, data on the effects of the dual use of cannabis and tobacco on health remains limited. Overall, further research is needed regarding the independent impact of cannabis on overall health, as well as the impact of dual use on health [24].

Here, we assessed whether current forms of cannabis are associated with deleterious health effects in young people, including poor diet and minimal exercise. Because of the high rates of dual use with nicotine and the potential for the combination of these drugs to cancel out dietary and exercise effects, we also investigated whether the dual use of nicotine and cannabis has effects on diet and exercise. Click-through rates are higher with short online surveys, and completion rates are tightly tied to length as well. Thus, we designed the shortest possible survey (goal of <10 questions that take <2 min to complete) to obtain critical data from our population of interest, including age, cannabis use, and tobacco use [25,26].

## 2. Materials and Methods

### 2.1. Survey Instrument and Distribution

We conducted an online, anonymous survey over five months, from December 2021 to April 2022, to gather data from young people 13–21 years of age who gave informed consent (those aged 18 years or older) or assent (those <18 years of age). The survey instrument was distributed across social media platforms, including Reddit, Facebook, Instagram, and Twitter platforms. The survey was posted four times a week across all four sites by two separate team members.

This survey, entitled “Teenage General Health and Marijuana Use”, was distributed online and programmed with SurveyMonkey. It consisted of 8 core questions, which assessed self-reported demographic variables (age, gender, race, and ethnicity), smoking tobacco or vaping nicotine-containing e-cigarettes (with options of never, rarely, monthly, weekly, or daily use), and past and current cannabis use (options included edibles, smoked, vaped, or other), using questions adapted from the UCSD Inhalant Survey (Table 1) [27,28,29]. Questions about diet and exercise were derived from standardized surveys, with the exercise question designed from the Stanford Leisure-Time Activity Categorical Item with consolidation down to five answer options [30]. The diet question was derived from the 2001–2002 Health Behavior in School-Aged Children survey, with conversion into one matrix question. The subjects who reported current or prior cannabis use were given 3 additional questions in order to define the frequency of use and specific forms of cannabis used. Because online, social media-based survey click-through and completion rates are tightly tied to length (shorter surveys have higher click-through and completion rates), more detailed questions regarding cannabis and tobacco use were not included [25,26]. This study was approved by the institutional review board of the University of California San Diego.

### 2.2. Survey Responses

**Figure 1:**
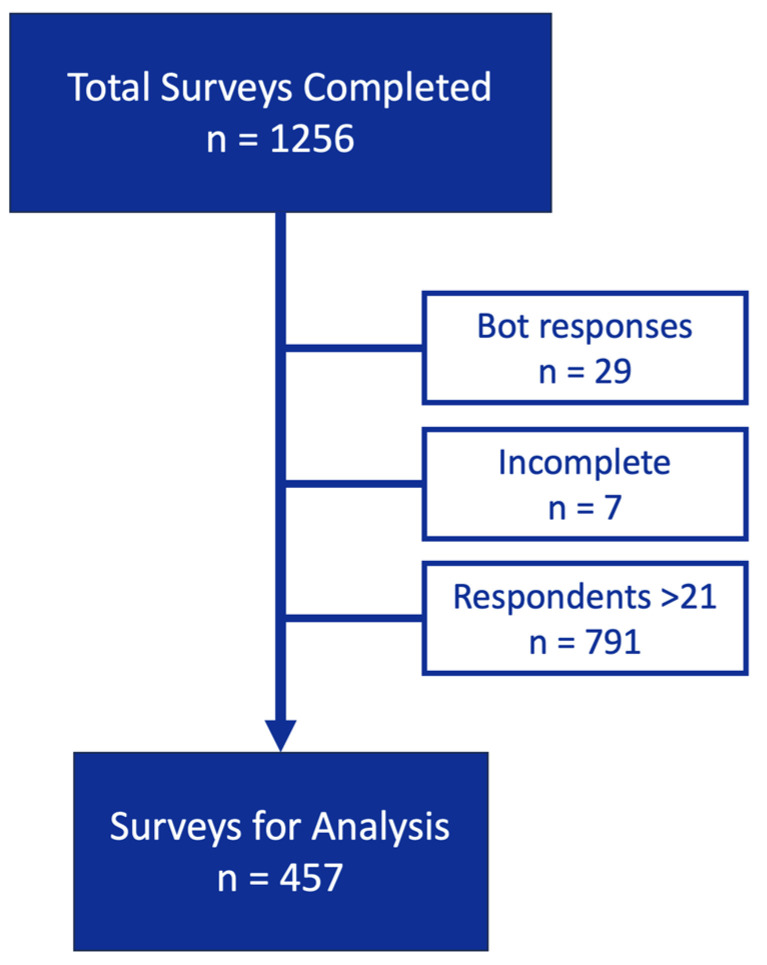
Surveys included in the analysis.

A total of 1256 surveys were completed and collected. Each participant who provided a valid email address was entered into a weekly lottery for a $250 Amazon gift card. The subjects were assigned a number, and the winners were selected through a random, blinded process using a random number generator. After the removal of computer generated (“bot”) responses (n = 29), incomplete responses (n = 7), and responses from subjects >21 years of age (n = 791), 457 surveys remained for analysis (Figure 1). The responses were categorized into four groups: controls (never or prior cannabis use), active cannabis users, nicotine users, and dual users (cannabis + the use of any form of inhaled nicotine).

### 2.3. Analysis

Age was analyzed as a continuous numeric variable, with ages 13–17 years combined because some groups had no subjects of these ages, across the four groups using Chi-squared tests. Gender (non-binary, gender variant/non-conforming, and prefer to self-describe were combined), race (prefer not to answer was combined with prefer to self-describe), and ethnicity were analyzed as nominal categorical variables across the four groups using Chi-squared tests (R version 4.4.1). Rcompanion (version 2.4.36) was used for post hoc analyses, using the pairwiseNominalIndependence function, with Bonferroni correction for multiple comparisons [31]. Diet and exercise habits were scored, with a lower score corresponding to a less healthy lifestyle. The diet and exercise data were analyzed as continuous numeric variables using the non-parametric Kruskal–Wallis test, with each group compared to the controls, and Dunn’s correction for multiple comparisons in GraphPad Prism version 10.3.0 (GraphPad Software, Boston, MA, USA). No outliers were identified or removed.

## 3. Results

### 3.1. Survey Responses

The online survey had a completion rate of 87%, with an average completion time of 2 min and 39 s, which accomplished our design goal of a short (<3 min) survey. Of the 457 teens and young adults who completed the survey, 44% reported active (within 30 days) cannabis use (n = 203; Table 2). The greatest number of participants were 21 years old (n = 120), with more active cannabis use in this age group (65%; n = 69). The majority identified as White (41%), Asian (34%), and Hispanic (23%; Table 2). Active cannabis users versus never/prior cannabis users were balanced on age, gender, race, and ethnicity.

**Figure 2:**
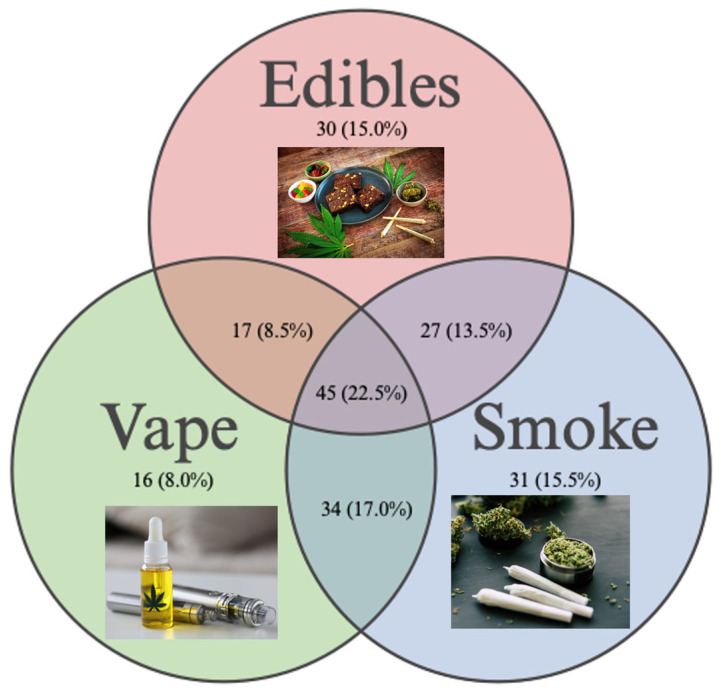
Cannabis use patterns. Created with BioRender [32,33,34].

Of the subjects reporting active cannabis use (n = 203; 44.4%), 14.8% (n = 30) reported using only edibles, 7.9% (n = 16) reported using only vape THC, 13.3% (n = 27) reported using edibles and smoking cannabis, 16.7% (n = 34) reported smoking and vaping cannabis, and 22.2% (n = 45) reported using edibles and smoking and vaping cannabis (Figure 2). Of the subjects reporting active tobacco use within the last 30 days (n = 95; 20.8%), the average age was 19.5 ± 1.3 years old, and 15.1% of females and 27% of males reported active tobacco use (Table 2). Of the tobacco users, 17.9% (n = 17) reported using only combustible cigarettes, 44.2% (n = 42) reported using only nicotine e-cigarettes, and 37.9% (n = 36) were dual users of both combustible cigarettes and nicotine e-cigarettes.

Sole users of cannabis represented 29% (n = 132), dual users of cannabis and nicotine e-cigarettes represented 7% (n = 31), dual users of cannabis and combustible tobacco represented 2% (n = 9), and triple users of cannabis, nicotine e-cigarettes, and combustible tobacco represented 7% (n = 31) of survey responders. Overall, dual users of both cannabis and nicotine represented 35% (n = 71) of active cannabis users and 16% of the overall cohort.

### 3.2. Diet and Exercise Habits

**Figure 3:**
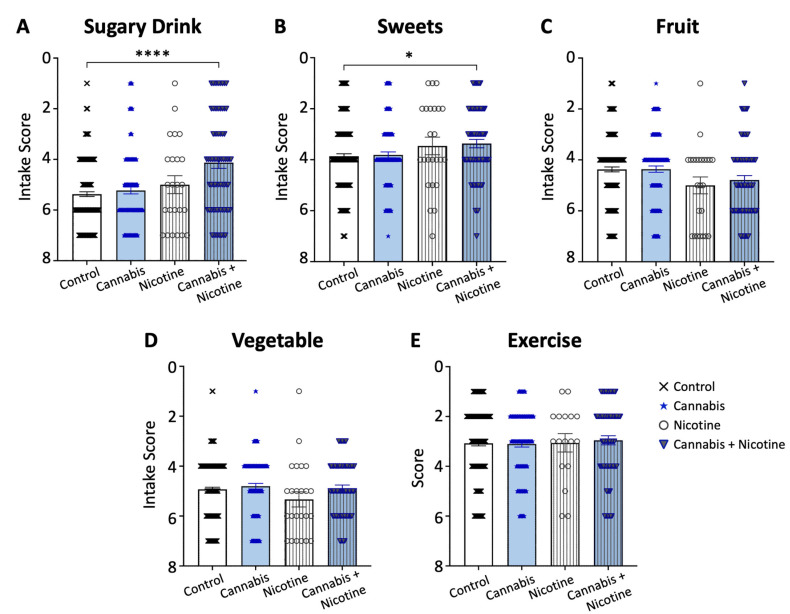
Dual users of cannabis and nicotine have higher dietary sugar intake. Sole users of cannabis reported similar intake to non-users across the food groups (**A**–**D**) and similar exercise levels (**E**). Dual users of both cannabis and nicotine reported higher intake of sugary drinks (**A**) and sweets (**B**). * *p* < 0.05; **** *p* < 0.0001.

Higher sugary drink consumption was associated with dual use of both cannabis and nicotine relative to the controls (with a mean of 3.87 and 2.63, respectively, *p* < 0.0001; Figure 3A), as well as an increased intake of sweets (with a mean of 4.63 and 4.12, respectively, *p* = 0.015; Figure 3B). We observed no significant association between active cannabis use or dual use with fruit and vegetable consumption relative to the controls (Figure 3C,D). Self-reported exercise patterns were similar between active cannabis users, dual users, and non-users (Figure 3E).

## 4. Discussion

We examined the effects of cannabis with and without concomitant nicotine use among young people aged 13 to 21 years and identified an association between the dual use of both and higher unhealthy sugar intake. This is concerning because unhealthy sugar intake, including both sugary beverages and sugary foods, is known to increase the development of diabetes and metabolic syndrome, which then confer an increased risk of cardiovascular disease. Populations disproportionately affected by tobacco, including Hispanic, BIPOC, and LGBTQIA+ communities, already have an increased risk of these diseases, which contributes to higher burdens of morbidity and mortality [18,35,36]. Identifying additional drivers or contributors to disease in these at-risk populations, such as the dual use of cannabis and tobacco products, may help to shape public policies to combat them.

Our finding that the combination of nicotine and cannabis use is associated with increased sugar intake is very intriguing. In terms of the potential mechanisms, studies have found that cannabinoid receptor 1 (CB1) activation via THC binding reduces the release of the gamma-aminobutyric acid (GABA) neurotransmitter and increases caloric intake, which is potentially the underlying mechanism of the “munchies” effect [37]. Contrastingly, nicotine has been shown to have hypophagic effects (suppression of caloric intake) due to increased serotonin (5HT) and dopamine (DA) in the lateral hypothalamic area [21]. Surprisingly, we did not observe any increase in dietary consumption with active cannabis use alone, which is contrary to the widely held theory that cannabis use drives higher food intake in general [3]. Instead, our data suggest that the combination of cannabinoid and nicotinic receptor activation in the central nervous system, which occurs during dual use, is required to promote hyperphagic effects for unhealthy sugary foods and drinks. We hypothesize that this may be a result of chemo-sensory changes promoting a desire for sugar intake. For example, the combination of nicotinic and cannabinoid receptor activation may diminish sweet receptors in the tongue or decrease the signaling associated with sugar intake. Alternatively, the dual use of both cannabis and tobacco products may be an indicator of unhealthy lifestyle choices in general.

Another potential mechanism by which dual use leads to increased sugar intake is via the effects of cannabis and tobacco on stress responses. Studies have shown that the co-administration of nicotine and THC increases c-Fos immunoreactivity in various areas of the brain, such as limbic and cortical structures, involved in the stress response [38]. Most of these areas are innervated by dopamine inputs, which suggests that the activation of mesolimbic and mesocortical dopaminergic systems may allow for interactions between nicotine and THC or cannabinoids. Stress levels are well-known to have a wide range of effects on diet. To further explore whether the increased sugar intake of dual users is associated with the stress-related effects of THC and nicotine co-administration, further studies are needed that include questions designed to quantify stress levels along with our diet and exercise questions.

We found no evidence of cannabis use being associated with less exercise, either by itself or in combination with forms of tobacco, which is consistent with recent studies in this area [8,9,39]. Tobacco and cannabis users had similar exercise levels compared to the control subjects, indicating that the use of either product is not associated with unhealthy lifestyle choices in the realm of physical activity.

Our study has multiple limitations. Because this was a social media-based survey, self-reporting bias, sampling bias, and participation bias may have impacted the data. Also, we are only able to infer correlations between drug use and other variables, not causation. We obtained responses from few nicotine-only users (cigarette smokers and e-cigarette vapers), such that we cannot draw conclusions about the impact of nicotine alone on diet and exercise. However, our findings raise questions that could be answered by future studies, where a larger sample size would allow for investigation of the roles of sex, gender, race, ethnicity, and socioeconomic status on dietary changes in the setting of cannabis and tobacco use, both together (dual use) and individually. Controlling for weight and BMI, or looking at interaction effects with BMI, would be important in future studies. Further, mechanistic studies to investigate the impact of cannabis use, with and without nicotine, on diet and exercise may be critical to fully understand the health effects of these drugs, as the effects of each drug individually may be disparate from their health effects in combination. Objective measures of health behaviors would strengthen future research, in addition to THC and cannabidiol potency measures.

## 5. Conclusions

Dual users of tobacco and cannabis have a higher intake of unhealthy sweet food and drink. These data suggest that dual users of tobacco and cannabis may develop metabolic syndrome at higher rates, which would subsequently increase the risk of cardiovascular complications. Further, our study confirms what many other groups have found, that young people have high rates of dual use of cannabis and tobacco and have high rates of using multiple forms of cannabis and tobacco. It is critical to elucidate the short and long-term effects of the combined use of these drugs, particularly because habits developed during this stage of life may have lifelong implications. Further research in this area will be critical to guide policy makers and increase awareness among the general population, as well as to improve the health of teens and young adults.

## Figures and Tables

**Table 1:** Cannabis and tobacco questions included on the UCSD Teen Cannabis and Tobacco Survey. Only the active use questions are shown. The past use questions were identical, but used past tense.

Inhalant Questions	Answer Choices
Are you actively using any form of THC, marijuana, or CBD products? Including but not limited to: THC pens, edibles, joints, cannabis oils, etc. *	Yes
	No
	Previous Usage
How many days per week do you currently use your THC products?	Sliding scale from “less than once a week” to “every day”
On days that you use THC, how many times do you typically use the product(s) throughout the day?	Sliding scale from “a couple times” to “a few times” to “constantly”
What forms of THC, cannabis, or CBD do you use? (choose all that apply)	Edibles
	Smoke
	Vape
	Other (please specify)
Have you ever used any of the following? (select: Never, Rarely, Monthly, Weekly, or Daily)	Cigarettes E-cigarettes

* Active use is defined as use within 30 days.

**Table 2:** Demographics of survey responders.

	Controls n = 230 (50.3%)	Cannabis Use n = 132 (28.9%)	Nicotine Use n = 24 (5.3%)	Cannabis + Nicotine Use n = 71 (15.5%)	Overall n = 457 (100%)	Chi-Squared Test *p* Value Post Hoc *p* Value
Age						** *p* = 0.004 ^$^ *p* = 0.001
13 ^#^	4 (1.7%)	0 (0%)	0 (0%)	0 (0%)	4 (0.9%)	
14 ^#^	5 (2.2%)	0 (0%)	0 (0%)	0 (0%)	5 (1.1%)	
15 ^#^	3 (1.3%)	0 (0%)	0 (0%)	0 (0%)	3 (0.7%)	
16 ^#^	4 (1.7%)	0 (0%)	0 (0%)	1 (1.4%)	5 (1.1%)	
17 ^#^	8 (3.5%)	3 (2.3%)	1 (4.2%)	4 (5.6%)	16 (3.5%)	
18	44 (19.1%)	13 (9.8%)	3 (12.5%)	16 (22.5%)	76 (16.6%)	
19	67 (29.1%)	32 (24.2%)	6 (25%)	12 (16.9%)	117 (25.6%)	
20	54 (23.5%)	35 (26.5%)	4 (16.7%)	18 (25.4%)	111 (24.3%)	
21	41 (17.8%)	49 (37.1%)	10 (41.7%)	20 (28.2%)	120 (26.3%)	
Average age:	19	19.9	19.8	19.4	19.4	
Gender						*p* = 0.10
Male	106 (46.1%)	62 (47.0%)	18 (75.0%)	44 (62.0%)	230 (50.3%)	
Female	112 (48.7%)	63 (47.7%)	5 (20.8%)	25 (35.2%)	205 (44.9%)	
Non-Binary ^#^	9 (3.9%)	4 (3.0%)	0 (0%)	1 (1.4%)	14 (3.06%)	
Gender Variant/	2 (0.9%)	2 (1.5%)	1 (4.2%)	0 (0%)	5 (1.09%)	
Non-Conforming ^#^						
Prefer to Self-Describe ^#^	1 (0.4%)	1 (0.8%)	0 (0%)	1 (1.4%)	3 (0.65%)	
Race						** *p* = 0.003 ^%^ *p* = 0.4 ^@^ *p* = 0.04
American Indian or Alaska Native	1 (0.4%)	2 (1.5%)	3 (12.5%)	3 (4.2%)	9 (1.97%)	
Asian	95 (41.3%)	36 (27.3%)	7 (29.2%)	15 (21.1%)	154 (33.7%)	
Black or African American	7 (3.0%)	6 (4.5%)	2 (8.3%)	7 (9.9%)	22 (4.81%)	
White or Caucasian	78 (33.9%)	60 (45.5%)	11 (45.8%)	36 (50.7%)	186 (40.7%)	
Mixed	19 (8.3%)	13 (9.8%)	1 (4.2%)	4 (5.6%)	36 (7.88%)	
Prefer not to answer ^#^	20 (8.7%)	9 (6.8%)	0 (0%)	1 (1.4%)	30 (6.56%)	
Prefer to self-describe ^#^	10 (4.3%)	6 (4.5%)	0 (0%)	5 (7.0%)	20 (4.38%)	
Hispanic or of Latino Descent						*p* = 0.7
Yes	47 (20.4%)	33 (25.0%)	7 (29.2%)	18 (25.4%)	105 (23%)	
No/Prefer not to Answer	183 (79.6%)	99 (75.0%)	17 (70.8%)	53 (74.6%)	352 (77%)	

^#^ Because some values were 0–1, these categories were combined for the Pearson’s Chi-squared test. ** *p* < 0.01. ^$^ Controls vs. Cannabis Use. ^%^ Control vs. Nicotine Use. ^@^ Control vs. Dual Use.

## Data Availability

Data is available upon request to the corresponding author.
